# COMPARATIVE EFFECTIVENESS RESEARCH FOR PROMOTING OLDER ADULT HEALTH: A SNAPSHOT OF PCORI-FUNDED PROJECTS

**DOI:** 10.1093/geroni/igae098.1462

**Published:** 2024-12-31

**Authors:** Regina Shih, Tabassum Majid

**Affiliations:** Chair; Co-Chair

## Abstract

The number of Americans over the age of 65 is projected to reach 95 million by 2060, making up approximately a quarter of the population. Health care, social, and community services can benefit from evidence-based strategies to better address the increasingly complex needs of this large and heterogeneous population. Comparative effectiveness research (CER) within these settings can promote older adult health when its patient-centered principles are embedded across the continuum of research from study design, conduct, evaluation, translation, implementation and dissemination with engagement throughout the process. PCORI’s approach to CER has resulted in a framework that addresses older adult health across the aging continuum. This session will describe three aging projects funded by PCORI: 1) healthy aging and villages, 2) dental care in low-income housing, and 3) comprehensive dementia care for patients and caregivers. PCORI program officers will kick off the symposium by describing its aging framework and funding priorities for real-world CER. The symposium will describe meaningful outcomes for older adults, their caregivers, evidence-based clinical practice, and the health, social, and community-based care systems that support older adults. Each presentation will feature the diverse perspectives of community, social service, or clinical partners on the projects and conclude with successes and challenges in conducting CER with older adults. This session will facilitate active discussion of approaches used across the continuum of aging and the research process with implications for future priorities in healthy aging interventions, caregiver supports, and integration of interventions into multiple care systems. Patient/Person Engagement in Research Interest Group Sponsored Symposium

